# Successful Nonoperative Treatment of a Lumbar Spine Extension Injury with Disruption of all Three Bony Columns in a Patient with Ankylosing Spondylitis – A Case Report

**DOI:** 10.2174/1874205X01711010092

**Published:** 2017-12-29

**Authors:** Ali Faqeeh, David Yen

**Affiliations:** Division of Orthopaedic Surgery, Department of Surgery, Queen’s University, Kingston, Ontario, Canada

**Keywords:** Lumbar spine, Ankylosing spondylitis, Brace, Inflammatory joint disease, Sarcoilial joints

## Abstract

**Study Design:**

A case report.

**Background:**

Patients with ankylosing spondylitis have altered spinal biomechanics putting them at increased risk of spinal fractures that are unstable. As a result there is an increasing trend to treat these fractures with surgical stabilization. We hypothesize that the fracture pattern is also an important factor in patients with this disease and that those with an extension injury in the lumbar spine can be treated with brace immobilization.

**Objective:**

Report on the non-operative management of an elderly patient, with ankylosing spondylitis, who sustained an extension injury of all three bony columns of the lumbar spine.

**Methods:**

A case report of a 70-year-old man who fell from a standing height, sustaining a three-column fracture at L1-2, who did not want surgical stabilization.

**Results:**

External brace immobilization was used and the patient was closely monitored. At his final 13 month follow-up, the patient had no clinical evidence of spinal instability or neurologic compromise and radiologically we could see callous formation anteriorly and laterally between the L1 and L2 vertebral bodies.These bridged the trebeculae across the middle and posterior columns at L1 and L2 on the lateral view, and there was no change in the sagittal or coronal alignment" to "There was mature bridging bone across the middle and posterior columns at L1 and L2 on the lateral view, and there was no change in the sagittal or coronal alignment

**Conclusion:**

This case supports our hypothesis that the fracture pattern is an important factor in patients with ankylosing spondylitis and adds to the body of knowledge in the scientific literature concerning non-operative treatment of fractures in patients with ankylosed spines. Further study is required to determine whether ours is an isolated case or whether this applies to a wider population of ankylosing spondylitis patients.

## 1 INTRODUCTION

Ankylosing spondylitis (AS) is a chronic inflammatory joint disease involving the spinal vertebra and the sacroiliac joints [1]. The mean prevalence of AS is 31.9 per 10,000 in North America [2]. Men are twice as likely to develop the disease compared to women, and 80% of cases develop AS before the age of 30 [3, 4].

The altered biomechanics of the spine in patients with ankylosing spondylitis increase the incidence of spinal fractures and the risk of subsequent displacement and neurologic injury [5, 6]. People with ankylosing spondylitis are four times more likely than the general population to sustain a spinal fracture, with a lifetime incidence ranging from 5% to 15% [7]. The vast majority of these are 3 column injuries resulting in an unstable spine [8].

The altered spine biomechanics with high risk of neurologic compromise result in an increasing trend towards surgical stabilization approaches to vertebral fractures in patients with AS [9-15]. However, these patients are at high risk of perioperative and operative complications [16-18] and therefore, non-surgical treatments, including bracing, traction, halo vest, and bedrest are used but typically only when surgical intervention would carry unacceptably high risk, or when the patient refuses surgical care [19]. Altun and Yuksel reported good results in18 patients treated conservatively with only one resultant pseudarthrosis. The authors concluded that nonsurgical treatment could be considered [20]. Kandziora advocates for prospective and perhaps randomized controlled studies comparing operative with conservative treatment in patients with ankylosing spinal disorders [21].

We report on a unique case of a patient with ankylosing spondylitis that sustained an extension injury of the lumbar spine (L1-2) with disruption to all three columns successfully treated with brace immobilization and followed with close and serial observation for 13 months.

## 2 CASE REPORT

A 70-year-old man presented to his local emergency department the day after sustaining a fall from a standing height. He complained of back pain but experienced no lower extremity numbness, weakness, or sciatica. On examination he was neurologically intact and ambulated without aids.

Plain film xrays showed a 3 column fracture at L1-L2 and ankylosis of the spine compatible with ankylosing spondylitis Figs. (**1**-**3**). Further investigation with a CT scan showed that the fracture was an AO type C1 extension injury (Fig. **4**).

Although surgery was discussed as a treatment option, the patient was not keen on operative stabilization of his fracture. In addition to the patient’s wishes, we also took into consideration his age and comorbidities of emphasema and peripheral vascular disease in deciding on nonoperative treatment. Therefore, he was instructed to wear a Jewett back brace while standing or walking. The patient was further instructed to avoid exercises and heavy or repetitive use of his back. His treatment plan was to monitor his progression, both clinically and with xrays, at 5 weeks, 3 months, 6 months, 9 months and 13 months.

When the patient presented over time at follow-up clinics, he noted a steady continuous improvement in his back pain and his ability to function. At 3 months he was independent in activities of daily living, and had switched from his back brace to using a lumbosacral corset. At 6 months he was increasing his mobilization and was able to resume playing darts. At 9 months he reported exercising at the gym 3 times per week. At this time he also reported performing his activities of daily living with ease.

**Fig. (1):**
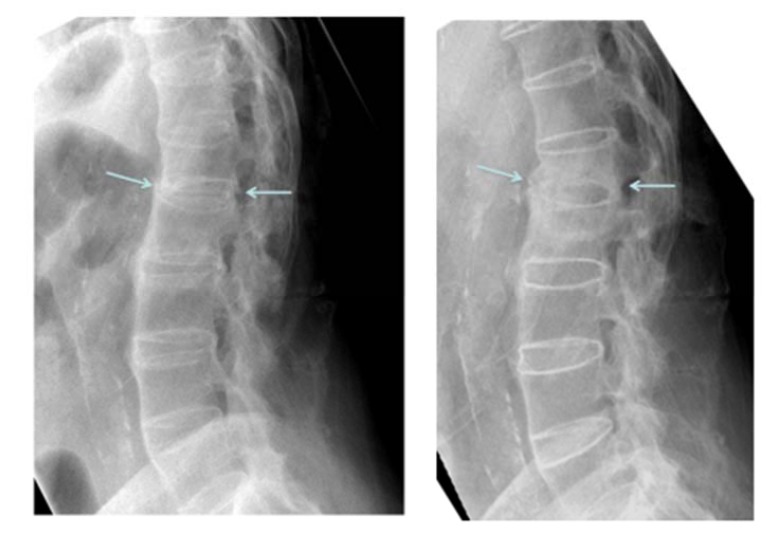
Arrows pointing to the anterior and middle column fracture areas on the initial lateral xray and follow up lateral xray.

**Fig. (3):**
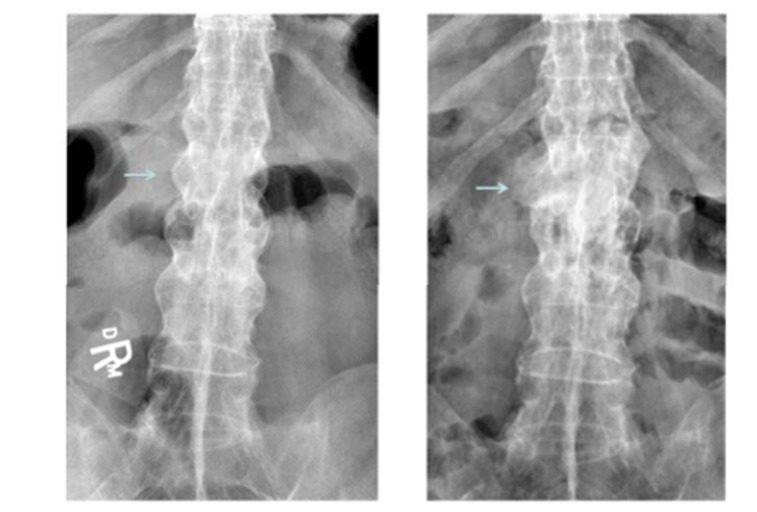
Arrows pointing to the fracture areas on the initial AP xray and follow up AP xray.

**Fig. (4):**
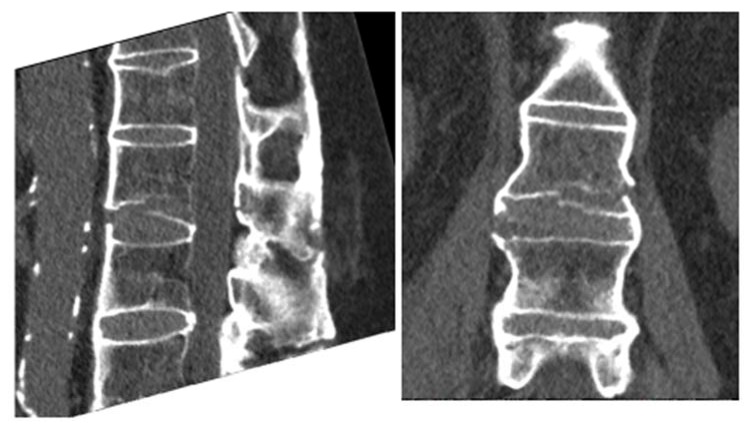
Initial CT scan sagittal and coronal reconstruction.

At the final follow-up visit which occurred at 13 months, the patient had discontinued the use of his brace. He had increased his activity and reported experiencing only minor and transient back pain after using a shovel. He had no signs or symptoms of neurologic dysfunction. At this time his x-rays showed callous formation anteriorly and laterally between the L1 and L2 vertebral bodies Figs. (**1** and **3**), mature bridging bone across the middle and posterior columns at L1 and L2 on the lateral view Figs. (**1** and **2**). There was no change in the sagittal or coronal alignments (Figs. **1** & **3**).

## 3 DISCUSSION

There is a move to operative over non-surgical treatment in AS patients with spine fractures due to the risk of subsequent displacement and neurologic injury [5]. Trent *et al.* [13] reported their experience with 7 AS patients over 8 years having thoracolumbar fractures. They concluded that shear injuries require surgery as soon as medically possible. However, none of their patients had extension injuries. Lu *et al*. [14] reported on 25 AS patients collected over 12.5 years, 14 treated surgically and 11 conservatively. They conclude that AS patients with unstable spinal fractures could benefit from early diagnosis and surgical treatment. However, none of their conservatively treated patients were extension type injuries in the lumbar spine. Westerveld *et al*. [9] reported on 14 AS patients collected over 7 years, 8 treated surgically and 6 conservatively. They concluded that surgical treatment may be beneficial for those with 3 column fractures. It is unclear whether any of the patients treated non-operatively had fractures in the lumbar spine. Whang *et al*. [15] reported on 12 AS patients over 6 years, 10 treated surgically and 2 conservatively. None of their 12 patient had fractures involving the lumbar spine. Altun and Yuksel [20] reported their experience with 30 patients over 13 years, 18 treated conservatively and 12 operatively. Only 1 of their patients had a lumbar fracture and they were treated operatively. Therefore, despite the authors’ enthusiasm for surgical treatment, it is not clear from the literature that non-operative management of extension injuries in the lumbar spine will not be successful.

Shen and Samartzis [22] have reported successful non-surgical treatment of a neurologically intact elderly patient with AS who sustained an unstable, three-column flexion-distraction injury at T5. The authors used this case to highlight the concept of a fourth column providing stability to the fractures spine, which is specific to the thoracic spine and consists of the rib-sternal complex. Our case is unique because the fracture was an extension injury and appeared in the lumbar spine (L1-2). Instead of a rib-sternal complex providing stability, we believe that the intact posterior paraspinal muscles acted as a hinge to prevent posterior opening and translation. This gave the fracture some stability and with added support from the brace, allowed the patient over time to return to full regular function without clinical or radiologic evidence of spinal instability or neurologic dysfunction. We consider this to be a successful outcome. To the best of our knowledge, ours is the first reported case with such a result in a neurologically intact AS patient who sustained an extension lumbar spine injury and was subsequently treated with brace immobilization.

## CONCLUSION

We describe a case of successful non-surgical management of an AS patient that sustained an extension injury of the lumbar spine with disruption to all three columns. This case supports our hypothesis that the fracture pattern is an important factor in patients with ankylosing spondylitis and that those with an extension injury in the lumbar spine can be treated with brace immobilization. Further research is required to determine whether ours is an isolated case or whether this applies to a wider population of ankylosing spondylitis patients.

## Figures and Tables

**Fig. (2):**
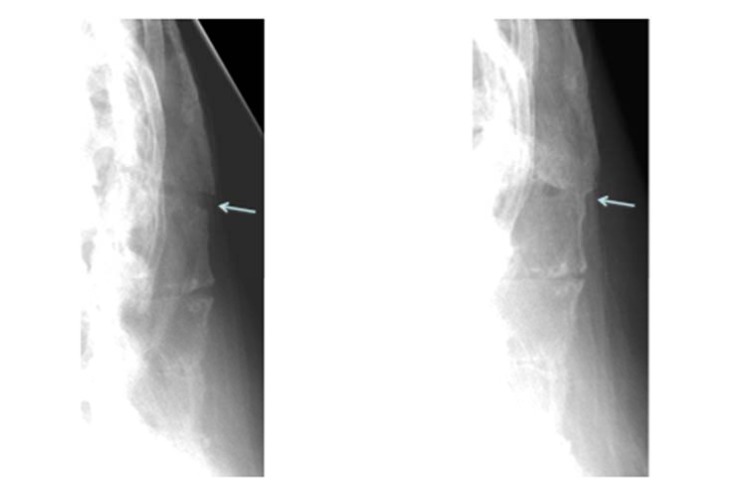
Arrows pointing to the posterior column fracture areas on the initial lateral xray and follow up lateral xray.
